# The spliceosome's secret life: *PRP8* silences transposons through 3D chromatin organization

**DOI:** 10.1093/plcell/koag231

**Published:** 2026-08-04

**Authors:** Satoyo Oya

**Affiliations:** Assistant Features Editor, The Plant Cell, American Society of Plant Biologists; Department of Plant Sciences, University of California Davis, Davis, CA 95616, United States; Department of Molecular Biology, Max Planck Institute for Biology Tübingen, Tübingen 72076, Germany

Forward genetics is a conversation with the plant, my PhD advisor used to tell me. You ask a question through a screen, and the plant answers through mutations. Their answers sometimes surprise you. In a new study, Qi, Zhuo, and colleagues (Qi et al., 2026) asked, “Which genes enforce silencing at a heavily DNA-methylated locus?” One unexpected gene came out: *PRP8*, the largest and most conserved component of the spliceosome (Grainger and Beggs, 2005).

DNA cytosine methylation silences genes and transposable elements (TEs), and how plants establish and maintain methylation is well characterized. What remains unclear, however, is precisely how methylation inhibits transcription. While several factors that enforce silencing on methylated loci have been identified, including *MOM1*, the *MORC* family ATPases, and the *MBD5/6–SLN* complex, the full cast of effectors that bridge the methyl mark to transcriptional repression remains incomplete.

To fill this gap, the authors exploited a forward genetic screen in a peculiar luciferase reporter line, where a luciferase gene is driven by a heavily methylated promoter yet remains moderately expressed, sustained by a balance between silencing and anti-silencing mechanisms. By knocking out the anti-silencing factor *HDP1*, the authors tipped this balance toward silencing, then screened for suppressor mutations that restore luciferase expression (Fig. 1a). This suppressor screen identified genes known to promote DNA methylation (*DRD1*, *NRPE1*, *AGO4*) and genes that act in silencing downstream of methylation (*MOM1*, *MORC1*, and *MORC6*). Unexpectedly, they also identified a missense mutation in *PRP8*, a core component of the spliceosome, and confirmed its causal role in *LUC* silencing through genetic complementation. The mutation in *PRP8* derepressed not only the luciferase reporter but also a set of endogenous loci known to be silenced through DNA methylation.

**Figure 1:**
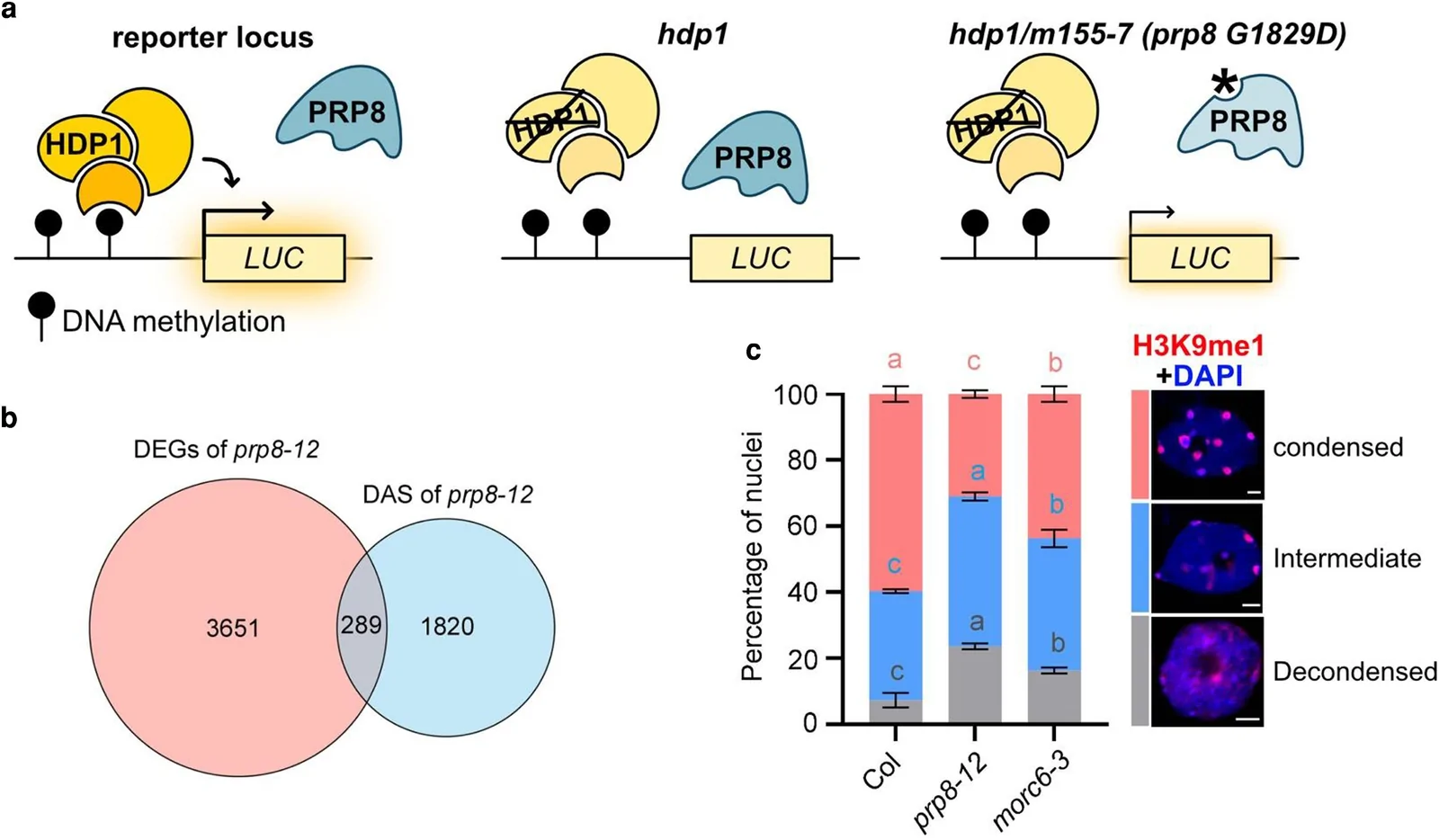
a) Schematic illustration of the YJ reporter system. b) Overlap between differentially expressed genes (DEGs, both up- and downregulated) and differentially alternatively spliced (DAS) genes in *prp8-12*. Of the ∼27,000 protein-coding genes annotated in Arabidopsis, the expected overlap assuming independence is ∼307 genes, close to the observed 289, suggesting that transcriptional derepression and splicing defects affect largely nonoverlapping gene sets but are not actively mutually exclusive. c) Quantification of condensed, intermediate, and decondensed chromocenter patterns in Col, *prp8-12*, and *morc6-3* nuclei. B and C are adapted from (Qi et al. 2026) Figures 7 and 5.

How does *PRP8* contribute to silencing? Although the *prp8* mutation causes widespread splicing defects, genes showing altered splicing did not significantly overlap with transcriptionally derepressed genes (Fig. 1b). Moreover, ChIP-qPCR showed PRP8 binding at derepressed TEs but no significant enrichment at genes with splicing defects, suggesting that silencing and splicing functions are independent.

Methylome analysis in the *prp8* mutant did not show a decrease in DNA methylation globally or at derepressed loci in any sequence contexts (CG, CHG, and CHH). Levels of the repressive histone marks H3K9me2 and H3K27me1, as well as siRNA accumulation, were similarly unaffected. *PRP8* thus appears to achieve transcriptional gene silencing (TGS) without altering the epigenetic marks known to be associated with TGS.

What pathway, then, does *PRP8* act through? One intriguing phenotype of *prp8* was alteration in the higher-order structure of chromatin. Pericentromeric heterochromatin, which is normally densely packed and appears as bright DAPI-spots known as chromocenters under the microscope, were visibly dispersed in *prp8* nuclei (Fig. 1c). This global loosening of the pericentromeric region seemed a robust phenotype, supported by genome-wide Hi-C analysis.

A natural question is how this phenotype relates to silencing. Because chromocenter decondensation is a hallmark of another TGS pathway gene, *MORC6*, the authors asked whether *PRP8* works in the same pathway. However, PRP8 showed no physical interactions with MORC6 or associated RdDM pathway factors, and genetic analysis revealed a synergistic effect of *prp8* and *morc6, and prp8* and RdDM mutants, indicating that these pathways are not perfectly redundant. Together, these results suggest that *PRP8* contributes to TGS through 3D chromatin organization, independent of its splicing activity and without converging on known methylation-dependent silencing pathways.

How PRP8 is recruited to the silencing target, and how it influences 3D genome organization and/or enforces silencing, remain fascinating open questions. As the authors note, most splicing factors previously linked to TGS in plants act through crosstalk with RdDM and influence DNA methylation; *PRP8* is a notable exception. The authors draw a parallel with *PRP31*, another spliceosomal factor implicated in methylation-independent TGS (Du et al. 2015), raising the possibility that noncanonical silencing functions may be more widespread than anticipated within the spliceosome. The Hi-C pattern observed in *prp8* resembles that reported by the same group under heat stress conditions, where TEs are activated without apparent changes in methylation (Sun et al. 2020), perhaps hinting at possible convergent mechanisms of the perturbed silencing and chromatin architecture.

How the spliceosome, 3D genome organization, and transcriptional silencing are wired together is a question this study opens as much as it answers—we will have to ask the plants again.

## Recent related articles in *The Plant Cell*

- Ren et al. (2024) shows that *MBD5* and *MBD6*, the methyl readers that recruit SLZ (Ichino et al. 2021) to silence methylated loci, also interact with MORC6 through an ACD-domain complex; whether *PRP8* intersects with this pathway remains an open question.
- Bazin et al. (2023) reports that 2 other spliceosome components, *PRP39a* and *SmD1b*, also promote gene silencing independently of their splicing activities—though at the posttranscriptional rather than transcriptional level.
- Kim et al. (2026) finds that the strength of interaction between STA1 and DOT2, 2 components of the same U4/U6·U5 tri-snRNP that harbors PRP8, regulates the formation of a subnuclear condensate known as nuclear speckles.

## Data Availability

No new data was generated for this In Brief article.
